# The Minimal Impact of Anthropogenic Disturbances on the Spatial Activities of Leopard Cats in Xinlong, China

**DOI:** 10.3390/ani13213328

**Published:** 2023-10-26

**Authors:** Xing Chen, Tengteng Tian, Han Pan, Yuyi Jin, Xiaodian Zhang, Qinggang Long, Ling Tang, Biao Yang, Li Zhang

**Affiliations:** 1 Key Laboratory for Biodiversity Science and Ecological Engineering, Ministry of Education, College of Life Science, Beijing Normal University, Beijing 100875, China; chenxing07mtx@163.com (X.C.); 202121200064@mail.bnu.edu.cn (T.T.); panhan_1996@163.com (H.P.); 2 College of Life Science, China West Normal University, Nanchong 637002, China; 3 Society of Entrepreneurs and Ecology (SEE) Foundation, Beijing 100020, China; anastasia0728@hotmail.com (Y.J.); xdz_daisy98@163.com (X.Z.); 4 China Environmental Protection Foundation, Beijing 100062, China; qingganglong@aliyun.com (Q.L.); 17341312391@163.com (L.T.)

**Keywords:** Mountains of Southwest China, small wild cat, livestock, conservation, coexistence

## Abstract

**Simple Summary:**

Human activities posed an increasing threat to leopard cats’ habitats. By utilizing infrared camera trapping, we analyzed the spatial distribution and habitat suitability of the leopard cats on Xinlong, located in the mountains of Southwest China. We also investigated the interaction between leopard cats and human disturbances in high-altitude regions. Our findings indicated that human disturbances had minimal effects on the habitat suitability, landscape structure, and spatial relationships of leopard cats. Their habitat preferences were shaped by competition with small carnivores like yellow-throated martens and environmental factors such as distance to water and terrain roughness index. This limited human impact may be attributed to local wildlife-friendly customs and leopard cat’s adaptability. To protect leopard cats and other wildlife, we recommend a conservation strategy that capitalizes on Xinlong’s distinctive ecological and cultural dynamics.

**Abstract:**

The habitat plays a crucial role in ensuring the survival of wildlife. However, the increasing disturbances caused by human activities present a substantial threat to habitats, especially for species such as the leopard cat (*Prionailurus bengalensis*), which is a significant small predator. Currently, research on leopard cats predominantly focuses on low-altitude regions within its distribution range, leaving plateau areas understudied. To enhance our understanding of the impact of human disturbances on leopard cat habitats, we undertook a study employing infrared camera trappings to monitor leopard cats’ activity in Xinlong of southwestern China between 2015 and 2023. We analyzed the spatial distribution and habitat suitability of the leopard cats by utilizing ensemble species distribution models (ESDMs). Moreover, we employed two-species occupancy models to investigate the spatial interaction between leopard cats and human disturbances. The results indicated that (1) the potential suitable habitat area for leopard cats encompassed approximately 1324.93 km^2^ (14.3%), primarily located along the banks of Yalong river. (2) The distribution of suitable habitat was predominantly influenced by competitors, specifically the yellow-throated marten (YTM), accounting for 52.4% of the influence, as well as environmental factors such as distance to water (DTW) at 12.0% and terrain roughness index (TRI) at 10.0%. Human interference, including cattle presence (4.6%), distance to road (DTD, 4.9%), and distance to settlement (DTS, 3.5%), had a limited impact on the habitat distribution. (3) Within a 5 km radius, habitat suitability increased with proximity to human settlements. (4) Leopard cats exhibited spatial independence from humans and domestic cattle (species interaction factor (SIF) = 1.00) while avoiding domestic horses (SIF = 0.76 ± 0.03). The relatively minor impact of human disturbances in Xinlong could be attributed to traditional cultural practices safeguarding wildlife and the leopard cat’s environmental adaptability. We recommend establishing a novel conservation paradigm based on the living dynamics of wildlife communities in Xinlong, thereby offering a more targeted approach to biodiversity preservation in the future.

## 1. Introduction

Habitat serves as the cornerstone for wildlife survival, offering essential elements like sustenance, breeding grounds, and refuge. However, human activities, notably urban expansion and infrastructure development, have triggered the escalating degradation and fragmentation of wildlife habitats on a global scale [1]. As a result of human intervention, the once-widespread presence of large carnivores has receded to eight major areas [2], with a mere 34% of these areas still hosting intact carnivore communities [3]. This decline in large carnivore populations has catalyzed the unlawful redirection of poaching activities toward smaller and medium-sized carnivores [4], leading to an imbalance in terrestrial ecosystems. In the current context, safeguarding global carnivore species has emerged as a foremost concern in contemporary conservation efforts [5,6,7].

The leopard cat (*Prionailurus bengalensis*), widely distributed across East and Southeast Asia [8], assumes a pivotal role within forest ecosystems as a keystone species, effectively regulating small mammal populations and upholding ecological equilibrium. However, the escalation of human activities in recent times has aggravated the fragmentation of the leopard cat’s habitat, leading to heightened concerns [9]. For instance, the invasion of human activities onto their habitats has facilitated the transmission of viruses from domestic cats to leopard cats [10]. Within China, many regions are witnessing a continued decline in leopard cat populations, with some even facing the risk of regional extinction [11]. The conservation of leopard cats urgently needs to be strengthened. Presently, leopard cats hold a Class II designation in *List of Key Protected Wild Animals in China* and are categorized as a vulnerable species in *China Biodiversity Red List* by the Chinese government. Nonetheless, due to the prevailing focus on flagship species under threat, smaller felids such as leopard cats have received comparatively less scholarly attention.

Thanks to its robust adaptability, leopard cats displayed a heightened capacity to accommodate various forms of human interference [12,13]. For example, in regions marked by human activity like oil palm (*Elaeis guineensis*) plantations, leopard cats strategically hunted rodents [14]. In the eastern distribution range of leopard cats, different regions showed varying strategies in response to human disturbances [11,15]. A survey of leopard cats in the Tai-hang Mountains, China, revealed that leopard cats tended to engage in relatively close-range activities in regions with moderate human interference, such as travel, transportation, farming, or grazing [11]. They also selected areas with slightly disturbed vegetation and demonstrated lower activity in more intensively disturbed zones [11]. A study on leopard cats in Taiwan found that they actively employed strategies to avoid humans [15]. They adjusted their activity areas in response to human activity levels, often preferring locations with higher invisibility [15]. Nonetheless, some evidence also suggested a potentially adverse influence of human intervention on leopard cats, encompassing the impact of domestic animals on both themselves and their prey [16]. Additionally, studies have pinpointed harvested forests (with greater openness) as more frequented by leopard cats [12]. As such, the effect of human interference on leopard cats appears multifaceted, necessitating further exploration to decipher the nuanced relationship between the two.

The mountains of Southwest China stands as one of the most ecologically intact terrestrial ecosystems globally, characterized by notable habitat diversity and well-rounded wildlife communities [17]. Positioned at the northwestern periphery of the leopard cat’s distribution range, this area exhibits distinctive environmental attributes compared to its eastern counterparts. Notably, the Chinese government’s initiatives, including the Natural Forest Protection Project and the Grain for Green Program, have shifted human-induced disruptions from deforestation and agricultural expansion to activities such as grazing and herb extraction [18]. Given the marked environmental contrast, it is probable that the behaviors of leopard cats in the mountains of Southwest China could significantly diverge from those in areas with serious human disturbances. However, it is worth noting that the leopard cat population in the mountains of Southwest China has received limited scholarly attention thus far.

In view of this, this study focused on Xinlong in Sichuan, situated within the mountains of Southwest China. Through investigating the spatial behaviors of leopard cats, this research aimed to elucidate the ramifications of local anthropogenic influences on habitat suitability and spatial dynamics, thereby contributing insights for the safeguarding and management of small carnivore populations.

## 2. Materials and Methods

### 2.1. Study Area

**Figure 1:**
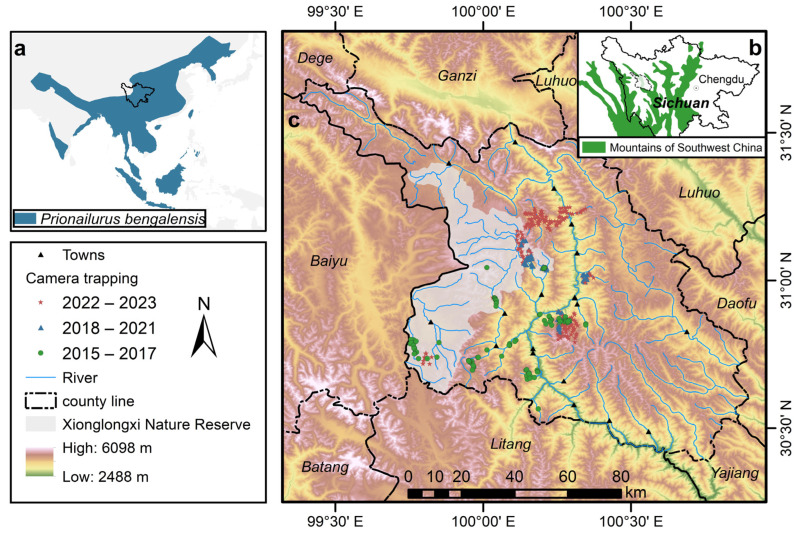
(**a**) The distribution of leopard cats (*Prionailurus bengalensis*); (**b**) the location of Xinlong in Sichuan province; (**c**) the distribution of camera trapping in Xinlong.

Xinlong, situated at the eastern edge of the Qinghai-Tibet Plateau (99°37′~100°54′ E, 30°23′~31°32′ N, Figure 1), spans from north to south along the Yalong river, giving rise to a canyon terrain characterized by significant topographical variations and an elevation range exceeding 3000 m. The climate is a subtropical humid monsoon mountain climate, featuring cold and arid winters and warm, rainy summers. Due to local cultural customs, the local people are very tolerant of wildlife [19], although there is a certain intensity of wild animal troubles in the local area [20]. Whether it is the diversity of carnivores, the diversity of habitats, or human disturbance, Xinlong is a representative research area in mountains of Southwest China which can more realistically restore the living conditions of carnivores.

### 2.2. Data Collection

#### 2.2.1. Species Distribution Data

We acquired species distribution data using infrared camera trapping from 2015 to 2023 (Figure 1c). Infrared cameras were strategically placed in areas frequented by wildlife, such as animal trails and streams. The cameras covered an altitude range of 3000–5000 m, spanning almost 2000 m and encompassing various vegetation types such as forests, scrublands, and meadows. The configuration of the infrared camera setup was detailed in the work by Tian et al. [21]. Leveraging the amassed infrared camera data, we collated the species’ points of occurrences and non-occurrences, subsequently filtering the points by using ArcGIS 10.5. Our approach involved the following steps: (1) we established a 1 km buffer zone around the presence points of leopard cats, removing non-presence points located within this buffer, and retaining only those points outside the buffer. (2) We further dilute both the presence and non-presence points within a 1 km screening range. When two or more points were found within this 1 km radius, we retained only one point to ensure the independence between points. Ultimately, the dataset employed for leopard cats’ analysis encompassed 62 occurrence points and 126 non-occurrence points. It is important to clarify that we categorize a point as a non-presence for a species if the species has not been detected by the camera during at least half a year of monitoring (camera days greater than 180).

#### 2.2.2. Environment Variables

In accordance with similar studies [22,23,24], we selected five environmental factors, including topography, vegetation, bioclimate and disturbance and biological factors to construct the pertinent model (Table S1). Topographic variables encompassed elevation (ELE), terrain roughness index (TRI) and distance to water (DTW), all extracted from the digital elevation model (DEM) of Xinlong (https://www.gscloud.cn/, accessed on 15 May 2023, resolution 30 m × 30 m) and calculated using ArcGIS 10.5 (Redlands, CA, USA. https://support.esri.com/, accessed on 15 May 2023). Enhanced vegetation index (EVI) served as the vegetation variable (https://data.tpdc.ac.cn/, accessed on 1 July 2023 [25]. EVI denotes variations in vegetation cover, with higher EVI values indicating increased vegetation cover. For wildlife, EVI can provide insight into the habitat’s concealment level to a certain extent. Bioclimatic variables comprised bio11 and bio19, sourced from Wordclim (https://worldclim.org/, accessed on 15 May 2023. Bio11 represents mean temperature of coldest quarter, bio19 represents the precipitation of the coldest quarter. The following disturbance variables were considered: distance to temple (DTT), distance to road (DTD), and distance to settlement (DTS). Road and settlement data were obtained from the National Catalogue Service for Geographic Information (https://www.webmap.cn/, accessed on 15 May 2023), while temple location data were provided by the Xinlong Forestry and Grassland Bureau. These variables were subjected to analysis via the Euclidean Distance tool in ArcGIS 10.5. It is worth noting that the inclusion of DTT as a variable was influenced by local cultural practices. Villagers residing near the temple engage in feeding activity towards wildlife, which could potentially impact the activity of animals. Hence, we choose this variable. Biological variables we considered included the site-specific photo rate we obtained during the study of large carnivores (including leopard (*Panthera pardus*) and wolf (*Canis lupus*)), small carnivores (red fox (*Vulpes vulpes*) and yellow-throated marten (*Martes flavigula*)), preys (white-eared pheasant (*Crossoptilon crossoptilon*), blood pheasant (*Ithaginis cruentus*), buff-throated partridge (*Tetraophasis szechenyii*) and woolly hare (*Lepus oiostolus*)), and livestock (domestic cattle and horse). Utilizing the aforementioned abiotic factors, we subsequently generated distribution probability layers for each species using subsequent analytical methods. Based on the model’s outcomes, we retained distribution layers for leopard, wolf, yellow-throated marten, white-eared pheasant, blood pheasant, buff-throated partridge, woolly hare, and domestic cattle. Distribution probability layers for each biological variable were calculated accordingly. In the case of large carnivores, the formula is *P*_carnivore_ = 1 − (1 − *P*_leopard_) × (1 − *P*_wolf_). *P*_carnivore_ represents the probability of at least one distribution of large carnivores, *P*_leopard_ represents the probability of distribution of leopards, and *P*_wolf_ represents the probability of distribution of wolves. Ultimately, we derived four biological variables: Carnivore (represents large carnivores, including leopard and wolf), YTM (represents small carnivore), Prey, Cattle (represents livestock).

We employed Spearman correlation analysis and Variance Inflation Factor (VIF) collinearity analysis to examine the interrelationships among environmental variables. When |r| > 0.7 and VIF > 2, we retained only one of the correlated variables. Ultimately, during the ensemble model analysis, we excluded two variables—ELE, due to its excessive correlation with YTM, and DTT, due to its pronounced correlation with Carnivores. The importance of ELE for wildlife habitat selection is self-evident. It should be noted that we excluded ELE and retained YTM because the model we built using ELE did not perform well, producing only one suitable model. In contrast, our analysis with YTM yielded better results. Consequently, we made the decision to eliminate the ELE variable from our study.

### 2.3. Data Analysis

#### 2.3.1. Ensemble Species Distribution Models

Species distribution models (SDMs) have gained increasing prominence in supporting conservation decision making [26]. Different modeling approaches exhibit distinct advantages and limitations [27,28,29], while ensemble species distribution models (ESDMs) offer the capability to amalgamate the strengths and drawbacks of diverse models, thereby enhancing the robustness of predictive outcomes. Within our study, our ensemble model encompasses a spectrum of models, namely generalized linear model (GLM) [30], generalized additive model (GAM) [31], generalized boosted regressions model (GBM) [32], classification tree analysis (CTA) [33], random forest (RF) [34] and maximum entropy model (MaxEnt) [35].

We choose cross-validation (holdout) to validate the ensemble model, and set 30% of the leopard cats’ presence records for model testing and 70% for training. We used 10 replicates for the cross-validation and used the average as the final result. We utilized the area under the receiver operating characteristic curve (AUC) [36] and Kappa [37] to gauge the predictive efficacy of our model results. Based on the AUC value, the results were classified as follows: <0.6, poor; 0.6~0.7, fair; 0.7~0.8, good; 0.8~0.9, fine; and >0.9, excellent [36]. Based on the Kappa value, the results were classified as follows: <0.2, poor; 0.21~0.40, fair; 0.41~0.60, good; 0.61~0.80, fine; and >0.9, excellent [37]. We applied a screening criterion for the models included in the ensemble model, setting an AUC threshold of >0.75.

The relative contribution of environmental variables was expressed as a percentage, and the ultimate outcome was derived from the weighted average of the ensemble model. The weight (W) was calculated as (AUC − 0.5)^2. The delineation of potential species habitats was established based on the threshold of the ensemble model’s final output. Within this study, the maximization of sensitivity and specificity (MSS) was selected as the threshold for distinguishing suitable and unsuitable habitats. The analysis was conducted employing the “SSDM” package within R 4.2.3 (https://www.r-project.org, accessed on 15 August 2023).

#### 2.3.2. Assessment of Habitat Landscapes

In order to further analyze the habitat status of leopard cats in Xinlong, we used the distribution results of potential suitable habitats for leopard cats obtained by the ensemble model to analyze the landscape pattern. We selected the following indices: number of patches (NP), patch density (PD), mean patch size (MPS), largest patch index (LPI), patch cohesion index (COHESION), landscape division index (DIVISION), splitting index (SPLIT), and perimeter–area fractal dimension (PAFRAC) (Table S2). The analysis was conducted using Fragstats 4.2 (https://fragstats.org/, accessed on 20 August 2023).

#### 2.3.3. Two-Species Occupancy Model

We applied two-species occupancy models to investigate the spatial interaction between leopard cats and human-induced factors (human, cattle, and horse). Following the prerequisites of occupancy modeling and the specifics of our dataset [38,39,40], we opted to employ monitoring data collected during the cold season (November to April of the subsequent year) for constructing the model. For each species, detection histories were established across 26 intervals, each spanning 7 days, corresponding to the duration of each camera deployment cycle. Camera monitoring time is too short, which may lead to a large error in model fitting. As a result, we excluded sites with fewer than 10 intervals (less than 70 days), ultimately yielding a total of 311 sites for the analysis. It is important to highlight that, due to the species’ behavior, complete independence between points cannot be guaranteed. Therefore, we interpreted the species’ occupancy rate as an indication of their habitat utilization.

Before embarking on the construction of two-species occupancy models, we subjected the environmental variables to a filtering process using single-species occupancy models. Within the single-species occupancy model, we incorporated the aforementioned environmental variables (abiotic factors) as occupancy covariates. Additionally, we also included Year as a covariate (Table S1). For the detection covariates, we chose to include Effort (representing camera days), Year, and DTT (Table S1). Ultimately, we constructed 176 models, including the null model, for each species. The model outcomes were ranked based on the Akaike information criterion (AIC). Subsequently, we selected the variables contained within the best-fitting model (with the lowest ΔAIC value) [41] to serve as the construction variables for the subsequent two-species occupancy models. For the analysis of the single-species occupancy models, we utilized the “unmarked” package within R 4.2.3 (Vienna, Austria).

The two-species occupancy model contained eight parameters [42] (Tables S3 and S4). Based on the model assumptions, human, cattle, and horse were considered the dominant species (species A) [16,43,44], and leopard cat was the subordinate species (species B) [11]. To avoid the non-convergence of the model, it is assumed that the detection of species A has no influence on the detection of species B at the same site (rBA = rBa) [45]. The model results were also ranked using the AIC. The model with the lowest AIC value was selected as the optimal model to extract the parameters and calculate the species interaction factor (SIF) estimates [42]. When SIF = 1, the spatial distributions of the two species were independent; when SIF was <1, the spatial distributions tended to be distinct; and when SIF was >1, the spatial distributions tended to overlap [42]. The two-species occupancy models were constructed using PRESENCE 2.13.47 (Reston, VA, USA. https://www.mbr-pwrc.usgs.gov/, accessed on 20 August 2023).

## 3. Results

Based on the model outcomes, we integrated the performance of GLM (AUC = 0.812, Kappa = 0.617), GBM (AUC = 0.771, Kappa = 0.595), GAM (AUC = 0.754, Kappa = 0.507), RF (AUC = 0.778, Kappa = 0.541), and MaxEnt (AUC = 0.758, Kappa = 0.516). Consequently, the ensemble model exhibited an AUC value of 0.7746 and a Kappa value of 0.5552. These results indicated that the model aligns with the “good” criterion, thus establishing its suitability for subsequent analyses.

### 3.1. Habitat Distribution

**Figure 2:**
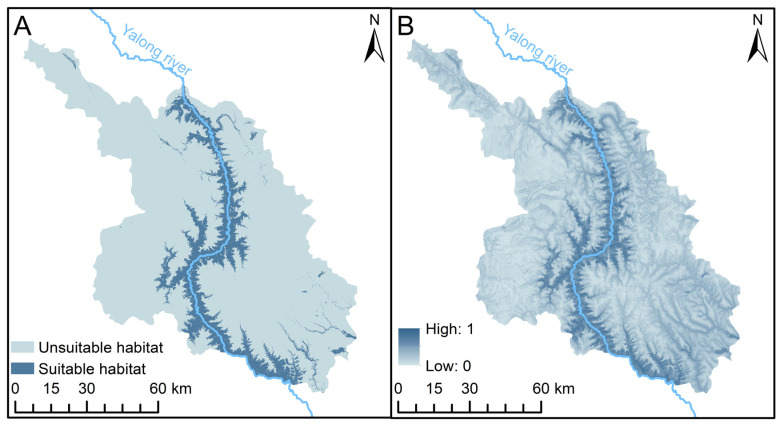
The distribution of suitable habitat for leopard cats in Xinlong of China by ensemble model. (**A**) Suitable and unsuitable habitat according to MSS (the maximizes the sum of sensitivity and specificity); (**B**) changes in the distribution of habitat suitability index.

Applying the established threshold (TSS = 0.3844), we partitioned the habitat distribution results into favorable habitats (>0.3844) and unfavorable habitats (<0.3844). The findings unveiled that leopard cats’ suitable habitat encompassed 1324.93 km^2^, constituting 14.25% of Xinlong’s total area (Figure 2A). Notably, these suitable habitats exhibited a predominant concentration within the forested regions flanking the Yalong river.

**Figure 3:**
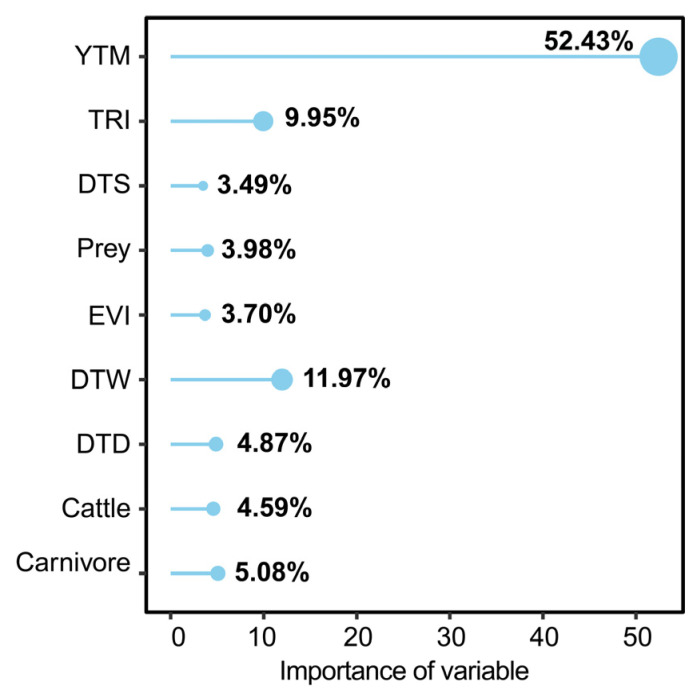
The importance of environmental variables affecting the spatial distribution of leopard cats. YTM represents small carnivores (yellow-throated marten), TRI represents terrain roughness index, DTS represents distance to settlements, Prey represents the probability of the presence of at least 1 species of prey (including white-eared pheasant, blood pheasant, buff-throated partridge and woolly hare), EVI represents enhanced vegetation index, DTW represents distance to water, DTD represents distance to road, Cattle represents livestock (cattle), Carnivore represents the probability of the presence of at least 1 species of large carnivores (including leopard and wolf).

**Figure 4:**
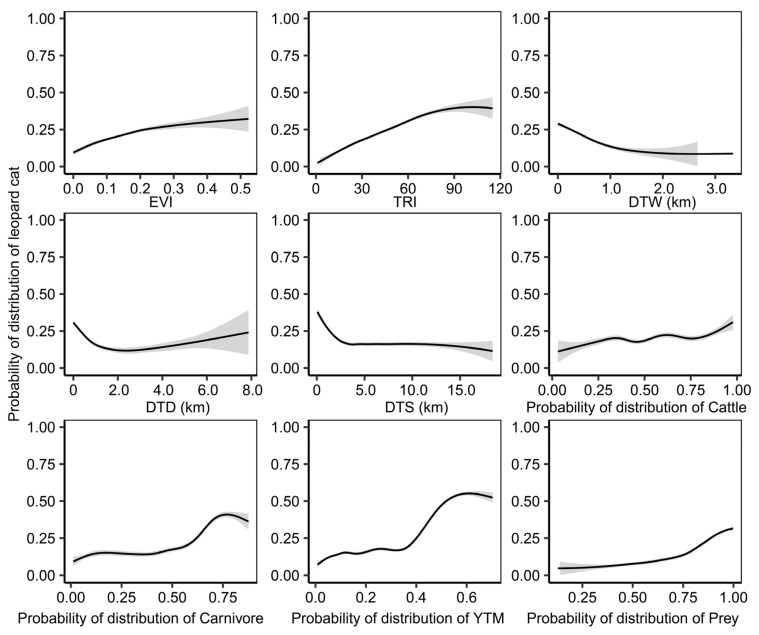
Effects of environmental variables on habitat suitability of leopard cats based on ensemble model. EVI represents enhanced vegetation index, TRI represents terrain roughness index, DTW represents distance to water, DTS represents distance to settlements, DTD represents distance to road, YTM represents small carnivores (yellow-throated marten), Carnivore represents the probability of the presence of at least 1 species of large carnivores (including leopard and wolf). Prey represents the probability of the presence of at least 1 species of prey (including white-eared pheasant, blood pheasant, buff-throated partridge and woolly hare).

Our assessment of model variable importance yielded the following insights: (1) The variables small carnivores/yellow-throated marten (YTM) demonstrated the highest significance at 52.4%, with leopard cats’ habitat suitability index exhibiting a positive correlation with YTM. (2) The next was the variable of distance to water (DTW), accounting for 12.0%, with habitat suitability increasing in proximity to water sources. (3) The variables of distance to road (DTD), Cattle (represents livestock), and distance to settlement (DTS) registered importance of 4.9%, 4.6%, and 3.5%, respectively (Figure 3 and Figure 4). Overall, the findings underscored that the spatial distribution of leopard cats was primarily influenced by competitive interactions and environmental factors, with human disturbance exerting minimal impact on leopard cats.

### 3.2. Landscape Patterns of Potential Habitats

The results showed that (Table 1) (1) the NP of the suitable habitat of leopard cat was 6903, PD was 0.7426 ind./km^2^, MPS was 19.19 km^2^, and LPI was 13.1%. This indicated that the habitat fragmentation was low, and the habitat was relatively complete. (2) COHESION was 99.9%, DIVISION was 0.98, and SPLIT was 58.24. This indicated that the degree of habitat aggregation was high. (3) PAFRAC was 1.4656, suggesting that the habitat had experienced human disturbance, albeit not to a significant extent.

### 3.3. Spatial Interactions

The results from the single-species occupancy models for both leopard cats and human disturbance (Table S5) indicated that the covariates affecting occupancy in the optimal model were terrain roughness index (TRI), DTW, elevation (ELE), and Year for leopard cats; DTD and Year for human; enhanced vegetation index (EVI) for cattle; and EVI and Year for horse. These environmental variables were included in the analysis of the respective two-species occupancy models and the results are presented in Table S6. The results showed that, the spatial distribution of leopard cats and horse tends to be separated (SIF = 0.76 ± 0.03), while they maintained independence from human and cattle (SIF = 1).

## 4. Discussion

The impacts of human disturbance on wildlife have been extensively documented in numerous studies [2,46,47]. Despite their critical ecological role, small carnivores have often received less attention compared to their larger counterparts [48]. Yet, these smaller carnivores face heightened vulnerabilities in their environment and encounter a more pronounced array of threats [49], thus underscoring the urgency of their enhanced protection. The mountains of Southwest China are an ecological treasure renowned for its relative absence of substantial human disruption [17,50]. Our study revealed that the spatial behaviors and habitat suitability of leopard cats in Xinlong were primarily influenced by the natural environment and competitors. Consequently, the contribution of human interference appears relatively minor. Despite a smaller habitat extent, the potential habitat quality for leopard cats remains elevated, accompanied by minimal fragmentation.

Prior investigations have demonstrated the leopard cats’ broad distribution and their capacity to adapt across diverse habitats such as meadows, forests, and shrubs [8]. Our findings further illuminated that the leopard cats’ suitable habitat predominantly congregates within the forested expanse flanking the Yalong river, with a notable absence in the alpine meadow zone. This aligns harmoniously with the prevailing consensus that leopard cats exhibit a penchant for understory activities [12]. Simultaneously, our analyses underscored that the foremost influence dictating the distribution of suitable habitats for leopard cats was the presence of competitors, particularly yellow-throated martens. Significantly, the impact of human interference was overshadowed by this competition dynamic. While our findings indicated a positive correlation between the distribution probability of leopard cats and YTM, it does not necessarily imply a positive impact of yellow-throated martens on leopard cats. We speculated that both species may prefer understory activities. Additionally, our results from another study revealed that the spatial distribution of leopard cats and small- and medium-sized carnivores, including yellow-throated martens and red foxes, were independent (SIF = 1), and overlapped with leopards (SIF = 1.17 ± 0.1) [51]. Therefore, our results suggest that the spatial behavior of leopard cats was heavily influenced by a strategic consideration of competition posed by small- and medium-sized carnivores [52,53].

The predominant human-induced disruptions observed in Xinlong encompassed grazing and herb-digging activities [21]. Examination of the environmental response curve unveils a gradual increment in leopard cats’ habitat suitability as the probability of cattle activity increases (Figure 4). Furthermore, within a 5 km radius of settlements, habitat suitability exhibited an upward trend with proximity to these human habitation areas (Figure 4). In human-populated regions, a potentially elevated rodents’ presence might facilitate leopard cats’ access to prey, thereby encouraging their movement toward areas closer to settlements [14]. The results suggested an adept adaptability of leopard cats to the presence of human interference. Notably, Xinlong, serving as a traditional gathering place for the Tibetan community in China, bears the profound influence of Buddhist principles emphasizing “no killing,” thereby significantly shaping local practices and lifestyles. This ingrained ethos among the local populace contributes to their notably amicable disposition toward wildlife.

Nonetheless, the results of the two-species occupancy model reveal a tendency for the spatial distribution of leopard cats and horses to diverge, maintaining independence from cattle, thereby implying that grazing activities exert some influence on leopard cats. Similar findings from studies conducted in the eastern region of leopard cat habitats also highlight the negative impact of livestock activity on the movement of leopard cats and their prey species [16]. Livestock, through trampling and foraging, can disrupt rodents’ habitats [54], thereby causing a decline in small mammal populations [55]. In Xinlong, the population of domestic cattle (142,744 in 2020) far surpasses that of horses (14,325 in 2020) (http://www.gzz.gov.cn/, accessed on 25 August 2023). Curiously, we observed that leopard cats demonstrated a greater inclination to avoid horses. This observed behavior might stem from the varying environmental repercussions associated with cattle and horses [56]. Horse digestion is not as thorough as that of cattle, leading to a more potent odor in their excrement, which could potentially exert a greater impact on wildlife. The multifaceted interplay between livestock and wildlife warrants further exploration, given its intricate and nuanced nature.

## 5. Conclusions

Presently, wildlife research concerning the impact of human disturbance has primarily revolved around flagship species like giant panda (*Ailuropoda melanoleuca*) [57] and large carnivores [58], often sidelining smaller carnivores. Xinlong emerges as a distinctive locale, intertwining natural and cultural assets that safeguard the cohesiveness of its wildlife community and establish it as research setting capable of accurately capturing genuine wildlife behaviors. Our findings underscored that leopard cats’ survival appears less influenced by lower levels of human interference intensity. Remarkably, the disturbances within Xinlong exhibited negligible impact on leopard cats’ habitat suitability, landscape configuration, and spatial interactions. In terms of the broader wildlife community, Xinlong features an array of predators capable of jeopardizing leopard cats’ existence, including leopards, wolves, lynxes (*Lynx lynx*), red foxes, Asiatic golden cats (*Catopuma temminckii*), and yellow-throated martens [59]. Intriguingly, leopard cats’ activities appeared to be more responsive to other predators than to human presence [58]. The amalgamation of Xinlong’s natural environs and indigenous cultural practices in wildlife conservation offers a beacon of hope for biodiversity conservation. Consequently, we advocate for the establishment of a novel conservation paradigm grounded in the realities of wildlife communities in Xinlong, a step that holds the potential to steer biodiversity protection towards a more promising trajectory.

## Figures and Tables

**Table 1:** The landscape pattern analysis results of leopard cat’s habitat in Xinlong, China.

Landscape Index		Leopard Cats’ Habitat	
		Suitable	Unsuitable
Fragmentation	Number of patches, NP	6903	8782
	Patch density, PD (ind./km^2^)	0.7426	0.9448
	Largest patch index, LPI (%)	13.1019	42.4762
	Mean patch size, MPS (km^2^)	19.1935	90.7582
Connectivity	Patch cohesion index, COHESION (%)	99.8979	99.9644
	Landscape division index, DIVISION	0.9828	0.644
	Splitting index, SPLIT	58.2374	2.8087
Human disturbance	Perimeter–area fractal dimension, PAFRAC	1.4656	1.4181

## Data Availability

The data presented in this study are available on request from the corresponding author.

## Supplementary Materials

The Following supporting information can be downloaded at https://www.mdpi.com/article/10.3390/ani13213328/s1. Table S1: Description of environmental variables in the models; Table S2: Landscape index description used in the landscape analysis; Table S3: Parameters’ description used in the conditional two-species occupancy model; Table S4: The candidate models of two-species occupancy model; Table S5: The results of covariates choosing for each species by using single-species occupancy model; Table S6: The results of two-species occupancy model for leopard cat and human disturbance.
