# Supplementary material for: The Minimal Impact of Anthropogenic Disturbances on the Spatial Activities of Leopard Cats in Xinlong, China
Source: Animals (Basel). 2023 Oct 26;13(21):3328. doi: 10.3390/ani13213328 (PMC10650319; doi:10.3390/ani13213328)
Supplement: Supplementary file 1 [file animals-13-03328-s001.zip › animals-2618098-supplementary.pdf]

Table S1 Description of environmental variables in the models

Variable	Code	Description	Types	Models
Vegetation factor	EVI	Enhanced vegetation index	Continuous	Ensemble model; occupancy model
Topographic factor	ELE	Elevation	Continuous	Occupancy model
	DTW	Distance to water	Continuous	Ensemble model; occupancy model
	TRI	Terrain roughness index	Continuous	Ensemble model; occupancy model
Disturbance factor	DTD	Distance to roads	Continuous	Ensemble model; occupancy model
	DTT	Distance to temple	Continuous	Occupancy model
	DTS	Distance to settlements	Continuous	Ensemble model
	Cattle	Probability of Cattle	Continuous	Ensemble model
Biological factor	Carnivores	Probability of Carnivores	Continuous	Ensemble model
	YTM	Probability of Yellow-throated marten	Continuous	Ensemble model
Bioclimatic factor	bio11	Mean temperature of coldest quarter	Continuous	Occupancy model
	bio19	Precipitation of coldest quarter	Continuous	Occupancy model
Others factor	Effort	Camera days	Continuous	Occupancy model
	Year	16-17; 19-20; 20-21; 22-23	Categorical	Occupancy model

Table S2 Landscape index description used in the landscape analysis.

**Landscape index**	**Description**
Number of patches (NP)	NP stands for the count of landscape patches within a specific category, with a higher NP signifying increased fragmentation.
Patch density (PD, ind./km^2)	PD represents landscape heterogeneity and fragmentation overall, where higher values signify heightened landscape fragmentation and spatial heterogeneity.
Largest patch index (LPI, %)	LPI serves as an indicator of the predominant patch's dominant influence within the landscape.
Mean patch size (MPS, km^2)	MPS can quantify landscape fragmentation.
Patch cohesion index (COHESION, %)	COHESION equals 1 minus the sum of patch perimeter (in terms of number of cell surfaces) divided by the sum of patch perimeter times the square root of patch area (in terms of number of cells) for patches of the corresponding patch type, divided by 1 minus 1 over the square root of the total number of cells in the landscape, multiplied by 100 to convert to a percentage.
Landscape division index (DIVISION)	DIVISION equals 1 minus the sum of patch area (m^2) divided by total landscape area (m^2), quantity squared, summed across all patches of the corresponding patch type.
Splitting index (SPLIT)	SPLIT equals the total landscape area (m^2) squared divided by the sum of patch area (m^2) squared, summed across all patches of the corresponding patch type.
Perimeter-area fractal dimension (PAFRAC）	PAFRAC assesses landscape shape complexity and, to some extent, signifies the extent of human influence on landscape patterns. A value nearing 1 suggests limited human impact, while a value approaching 2 indicates a higher degree of influence from human activities

Table S3 Parameters description used in the conditional two-species occupancy model

Parameter	Description
*psi*A	Probability of occupancy for species A
*psi*BA	Probability of occupancy for species, given species A is present
*psi*Ba	Probability of occupancy for species, given species A is absent
*p*A	Probability of detection for species A, given species B is absent
*p*B	Probability of detection for species B, given species A is absent
*r*A	Probability of detection for species A, given both species are present
*r*BA	Probability of detection for species B, given both species are present and species A is detected
*r*Ba	Probability of detection for species B, given both species are present and species A is not detected

Table S4 The candidate models of two-species occupancy model

Model type	Hypothesis
*psi*A, *psi*B; *p*A, *p*B	Neither environment nor species A presence influences species B's occupancy/detection
*psi*A, *psi*B; *p*A, *p*B, *r*B	Only species A presence influences species B's detection
*psi*A, *psi*BA, *psi*Ba; *p*A, *p*B	Only species A presence influences species B's occupancy
*psi*A, *psi*BA, *psi*Ba; *p*A, *p*B, *r*B	Only species A presence influences species B's detection/occupancy
*psi*A (H), *psi*B (H); *p*A , *p*B	Only environment influences species B's occupancy/detection
*psi*A (H), *psi*B (H); *p*A , *p*B , *r*B	Environment-mediated influence of species A presence on species B's detection
*psi*A (H), *psi*BA (H), *psi*Ba (H); *p*A , *p*B	Environment-mediated influence of species A presence on species B's occupancy
*psi*A (H), *psi*BA (H), *psi*Ba (H); *p*A , *p*B , *r*B	Environment-mediated species A presence influence on species B's detection/occupancy

Table S5 The results of covariates choosing for each species by using single-species occupancy model

Model	N	AIC	ΔAIC	AIC Wt
Prionailurus bengalensis				
p(effort+Year), *psi*(TRI+DTW+ELE+Year)	12	1218.87	0.00	0.07
p(effort+Year+DTT), *psi*(TRI+DTW+ELE+Year)	13	1219.50	0.63	0.05
p(effort+Year), *psi*(TRI+DTW+ELE+DTD+Year)	13	1220.72	1.85	0.03
p(effort+Year), *psi*(EVI+TRI+DTW+ELE+Year)	13	1220.83	1.96	0.03
**Human**				
p(effort+DTT), *psi*(DTD+Year)	8	2003.53	0.00	0.04
p(effort+Year+DTT), *psi*(EVI+DTD)	9	2004.01	0.49	0.03
p(effort+Year+DTT), *psi*(DTD)	8	2004.56	1.03	0.03
p(effort+DTT), *psi*(EVI+TRI+DTD+Year)	10	2004.61	1.08	0.03
p(effort+DTT), *psi*(DTT+EVI+DTD+Year)	10	2004.70	1.18	0.02
p(effort+DTT), *psi*(EVI+bio19+DTD+Year)	10	2004.94	1.42	0.02
p(effort+DTT), *psi*(EVI+ELE+DTD+Year)	10	2005.06	1.54	0.02
p(effort+DTT), *psi*(EVI+DTW+DTD+Year)	10	2005.17	1.64	0.02
p(effort+DTT), *psi*(EVI+bio11+DTD+Year)	10	2005.18	1.66	0.02
**Cattle**				
p(effort+Year), *psi*(EVI)	7	2245.77	0.00	0.03
p(effort+Year), *psi*(EVI+DTD)	8	2245.80	0.03	0.03
p(effort+Year+DTT), *psi*(EVI)	8	2246.28	0.50	0.03
p(effort+Year+DTT), *psi*(EVI+DTD)	9	2246.34	0.57	0.02
p(effort+Year), *psi*(EVI+DTW)	8	2246.87	1.09	0.02
p(effort+Year), *psi*(EVI+ELE)	8	2247.29	1.52	0.02
p(effort+Year+DTT), *psi*(EVI+DTW)	9	2247.39	1.62	0.01
p(effort+Year), *psi*(DTT+EVI)	8	2247.58	1.80	0.01
p(effort+Year), *psi*(EVI+bio11)	8	2247.70	1.92	0.01
p(effort+Year), *psi*(DTD)	7	2247.71	1.94	0.01
p(effort+Year+DTT), *psi*(EVI+ELE)	9	2247.72	1.94	0.01
p(effort+Year), *psi*(EVI+TRI)	8	2247.75	1.98	0.01
**Horse**				
p(effort), *psi*(EVI+Year)	7	2278.88	0.00	0.04
p(effort), *psi*(DTT+EVI+bio11+Year)	9	2279.70	0.83	0.03
p(effort+Year), *psi*(EVI)	7	2279.81	0.93	0.03
p(effort+DTT), *psi*(EVI+Year)	8	2280.00	1.13	0.03
p(effort), *psi*(DTT+EVI+bio19+Year)	9	2280.46	1.59	0.02
p(effort), *psi*(DTT+EVI+bio11+DTD+Year)	10	2280.68	1.81	0.02
p(effort+DTT), *psi*(DTT+EVI+bio11+Year)	10	2280.71	1.84	0.02

Table S6 The results of two-species occupancy model for leopard cat and human disturbance

Model	AIC	ΔAIC	AIC wgt	Model Likelihood	N	-2*LogLike
**Human-leopard cat**						
psiA(DTD+Year), psiB(TRI+DTW+ELE+Year); pA, pB, rB	3418.78	0	0.6346	1	14	3390.78
psiA(DTD+Year), psiB(TRI+DTW+ELE+Year); pA, pB	3420.66	1.88	0.2479	0.3906	14	3392.66
psiA(DTD+Year), psiBA(TRI+DTW+ELE+Year), psiBa(TRI+DTW+ELE+Year); pA, pB, rB	3422.94	4.16	0.0793	0.1249	18	3386.94
psiA(DTD+Year), psiBA(TRI+DTW+ELE+Year), psiBa(TRI+DTW+ELE+Year); pA, pB	3424.4	5.62	0.0382	0.0602	18	3388.4
psiA, psiBA, psiBa; pA, pB, rB	3512.38	93.6	0	0	8	3496.38
psiA, psiB; pA, pB, rB	3512.4	93.62	0	0	8	3496.4
psiA, psiBA, psiBa; pA, pB	3514.01	95.23	0	0	8	3498.01
psiA, psiB; pA, pB	3514.56	95.78	0	0	8	3498.56
**Cattle-leopard cat**						
psiA (EVI), psiB (TRI+DTW+ELE+Year); pA , pB	3619.43	0	1	1	13	3593.43
psiA (EVI), psiB (TRI+DTW+ELE+Year); pA, pB, rB	3653.17	33.74	0	0	13	3627.17
psiA (EVI), psiBA (TRI+DTW+ELE+Year), psiBa (TRI+DTW+ELE+Year); pA, pB, rB	3653.26	33.83	0	0	17	3619.26
psiA (EVI), psiBA (TRI+DTW+ELE+Year), psiBa (TRI+DTW+ELE+Year); pA, pB	3653.94	34.51	0	0	17	3619.94
psiA, psiBA, psiBa; pA, pB, rB	3674.62	55.19	0	0	8	3658.62
psiA, psiB; pA, pB, rB	3674.86	55.43	0	0	8	3658.86
psiA, psiBA, psiBa; pA, pB	3675.52	56.09	0	0	8	3659.52
psiA, psiB; pA, pB	3676.46	57.03	0	0	8	3660.46
**Horse-leopard cat**						
psiA (EVI+Year), psiBA (TRI+DTW+ELE+Year), psiBa(TRI+DTW+ELE+Year); pA, pB,rB	3611	0	0.3378	1	18	3575
psiA (EVI+Year), psiB (TRI+DTW+ELE+Year); pA, pB, rB	3611.21	0.21	0.3041	0.9003	14	3583.21
psiA (EVI+Year), psiBA (TRI+DTW+ELE+Year), psiBa(TRI+DTW+ELE+Year); pA, pB	3611.95	0.95	0.2101	0.6219	18	3575.95
psiA (EVI+Year), psiB (TRI+DTW+ELE+Year); pA , pB	3612.65	1.65	0.148	0.4382	14	3584.65
psiA, psiBA, psiBa; pA, pB, rB	3716.09	105.09	0	0	8	3700.09
psiA, psiB; pA, pB, rB	3716.1	105.1	0	0	8	3700.1
psiA, psiBA, psiBa; pA, pB	3717.23	106.2	0	0	8	3701.23

		3				
psiA, psiB; pA, pB	3717.57	106.57	0	0	8	3701.57
